# Quadriceps Muscle Morphology as a Marker of Performance Across Multiple Strength and Power Tests

**DOI:** 10.1155/tsm2/7599813

**Published:** 2026-07-18

**Authors:** Davi A. G. Mázala, Daniel F. McLaughlin, Matheus B. Guerrero, Líbyna T. C. Martins, Andrea T. Barton, Marcel B. Lanza

**Affiliations:** ^1^ Department of Kinesiology, College of Health Professions, Towson University, Towson, Maryland, USA, towson.edu; ^2^ Instituto de Matemática e Estatística, Universidade Federal de Uberlândia, Uberlândia Minas Gerais, 38.400-902, Brazil, ufu.br; ^3^ Department of Physical Therapy and Rehabilitation Science, University of Maryland School of Medicine, Baltimore, Maryland, USA, umaryland.edu

## Abstract

Previous studies demonstrated that measures of muscle thickness (MT) and echo intensity (EI) show an association with functional tests, but results have been inconsistent. The aim of the current cross‐sectional study was to determine which muscle from the quadriceps (QUAD) (i.e., vastus lateralis [VL], rectus femoris [RF], or vastus intermedius [VI]) and ultrasound measurements (i.e., MT and EI) better explain three different physical assessments: maximal isometric knee extension torque (MIVT), vertical jump peak power (VJPP), and lower limb muscle power (LLMP). Forty‐three adults (23 women) participated in this cross‐sectional study. Participants’ body composition, ultrasound measurements from the dominant leg, and functional outcomes were evaluated on the same visit. Bivariate analyses indicated moderate to strong positive associations between all physical assessments and muscle morphology variables, supporting the use of multiple regression analyses. Also, given the multicollinearity among EI measures, a global EI index was generated and used in the multiple regression analyses. The multiple regression analysis showed that MIVT was independently associated with RF MT (*p* = 0.009) and global EI (*p* = 0.004), with the model explaining 63.5% of the variance. For VJPP, RF MT was the only variable that significantly contributed to the model (*p* = 0.041), explaining 61.9% of the variance. Lastly, LLMP was independently associated with RF MT (*p* = 0.003) and VI MT (*p* = 0.010), and the model explained 63.0% of the variance. Overall, these findings suggest that RF MT may be the most informative QUAD ultrasound measure for explaining performance in our data set. This is particularly important in clinical and research settings in which reducing testing time, participant burden, and analytic complexity can improve feasibility without substantially compromising the relevance of the evaluation.

## 1. Introduction

Ultrasound was first introduced as an imaging modality in 1968 [1], and since then, improvements in technology have allowed for better quality resolution, thus leading to an increase in the number of research studies evaluating skeletal muscle characteristics via ultrasound. The two most commonly used metrics derived from ultrasound measurements are muscle thickness (MT) and echo intensity (EI) [2]. Ultrasound measurements of MT can represent a measurement of muscle size and have been shown to be reliable and comparable to magnetic resonance imaging (MRI) [3, 4]. EI has been widely used as a surrogate measure of muscle quality, and MRI‐derived intramuscular fat content has been shown to correlate with EI values. [5]. Both MT and EI are known important characteristics of skeletal muscle that are often changed due to aging, muscle disuse, or neuromuscular diseases [6, 7].

The quadriceps (QUAD) is one of the most important muscle groups for exercise performance due to its contribution to sprinting and jumping [8] and to day‐to‐day activities such as stair climbing [9] and standing from a seated position [10]. While multiple different tests can be used to evaluate QUAD function, the knee extension test is one of the most commonly used in research and clinical settings [11–13]. Indeed, QUAD muscle volume can explain 60% of maximal isometric voluntary contraction [14]. Yet, several studies have reported that knee extension isometric force production shows a weak relationship with dynamic performance [15–18], suggesting that functional tasks may better translate to sports performance and daily activities. Consequently, researches have examined other tasks such as jump [19, 20] and stair climbing ability [9, 21], though the influence of QUAD MT and EI on these tasks remains unclear. Stock et al. reported a significant correlation between the EI values of the vastus lateralis (VL) and rectus femoris (RF) with jump height in middle school athletes [2], whereas Laett et al. found no significant association between VL EI and jump performance in soccer players [22]. Similarly, Guevarra et al. showed that stair‐climb time, but not stair‐climb power, was correlated with QUAD MT in patients with symptomatic knee osteoarthritis [23]. In contrast, a study in career firefighters found that both QUAD MT and EI were associated with overall stair‐climb performance [9]. Overall, the inconsistency among studies highlights a significant gap in understanding how QUAD MT and EI contribute to functional performance.

A large number of studies using muscle ultrasound to evaluate the knee extensors have only focused on one muscle (i.e., VL or RF) [24–26], while omitting others such as vastus intermedius (VI) or vastus medialis (VM). The omission of the VM may be attributed to several considerations, including its location as well as anatomical size and shape, which make standardization of measurement location more challenging. Moreover, VM appears to present a relatively smaller contribution (∼9.5 to 24%) to total knee extensor torque compared to other QUAD muscles [27, 28]. Furthermore, roughly 63% of studies evaluating the upper leg have focused on either RF or VL [29], and only a limited number of investigators have also included other knee extensor muscles in their studies. For instance, Ando et al. evaluated the relationship between ultrasound measurements of MT from the VL, RF, VI, and VM with knee extension torque [13]. They showed that only the VI MT showed a significant correlation with knee extension torque; there were no correlations between VL, RF, and VM MT with knee extension torque [13]. Another study showed RF MT and EI were not associated with muscle strength, while the VL MT was associated with muscle strength, and both VI MT and EI were associated with muscle strength [30]. Additionally, we previously reported a significant overall relationship between EI values from the VL and RF and the rate of force development [31]. Overall, there are limited number of studies evaluating the correlation between MT and EI of different muscles from the QUAD with physical function. Thus, this suggests that more studies are needed to determine which muscle characteristic (i.e., MT and EI) shows the strongest association with physical function.

Nonetheless, understanding the importance of identifying the QUAD muscle that can best predict physical function is essential for clinicians and researchers. This might help to better inform our decision‐making process to more accurately and efficiently select the QUAD muscle and ultrasound parameter (MT and/or EI) to be evaluated, thereby reducing assessment time and improving the accuracy and applicability of ultrasound evaluations in clinical and research settings. Previous studies have evaluated the ability of different QUAD muscles to predict performance in different lab‐based tests, but results have not been consistent. Ando et al. showed that the VI MT was the best predictor of knee extension force production in healthy young men [13], while findings from Moreau et al. showed that the VL MT was the best predictor of knee extension torque in children and adolescents with and without cerebral palsy [32]. Others demonstrated that the VL cross‐sectional area was a predictor of Wingate test performance in amateur cycles [33], while the VI MT predicted the late phase of the knee extension rate of force development in resistance‐trained men [34]. In young boys, the VL MT was a predictor of rate of torque development while the VI MT was the best predictor of isometric knee extension and athleticism [2]. While these studies have evaluated the contribution of individual QUAD muscles to predict performance on different tests, only one of them examined more than two muscles of the QUAD [13]. Additionally, only one study evaluated the correlation between MT and EI with athletic performance and isometric strength [2]. Thus, more studies are needed to establish the contribution of different muscles from the QUAD as predictors of performance in multiple tests.

Therefore, the aim of this study was to determine which QUAD muscle (i.e., VL, RF, and VI) and ultrasound measurements (i.e., MT and EI) better explain the different physical assessment tests such as the maximal isometric knee extension torque (MIVT), vertical jump peak power (VJPP), and lower limb muscle power (LLMP). Considering the previous findings, we hypothesized that VL and/or VI would present the highest associations with physical performance, while MT rather than EI would show the greatest association with the different tests. Identifying the specific muscle and ultrasound parameter that best explains each test outcome will help streamline assessment protocols by allowing researchers and clinicians to focus on the most informative muscle and measurement. This is particularly important in clinical and research settings in which reducing testing time, participant burden, and analytic complexity can improve feasibility without substantially compromising the relevance of the evaluation.

## 2. Methods

### 2.1. Participants

Participants were recruited from the Greater Baltimore‐Washington, DC Area. This study was approved by the Towson University Institutional Review Board (IRB# 2048), and all procedures adhered to those outlined in the Declaration of Helsinki. Study inclusion criteria were the following: 18 to 45 years of age, healthy, and free of chronic disease or orthopedic injuries. Exclusion criteria for this study were as follows: individuals with known musculoskeletal or other injury that would prevent them from completing the exercise tests or with a history of surgical treatment in the area of interest for ultrasound measurements. Participants completed a self‐reported questionnaire about regular physical activity habits. A priori power analysis was initially conducted to determine the required sample size for the primary bivariate associations. Utilizing correlation values between maximum force and VL morphology from a previous study [31] (*r* = 0.505 for MT and *r* = −0.478 for EI), a required total sample size of 28–32 participants was estimated (two‐tailed bivariate normal model, alpha = 0.05, 1‐beta = 0.80). To ensure adequate recruitment and account for potential dropouts, 43 participants provided written informed consent. Because our analytical approach utilized multiple linear regression incorporating four structural predictors, a sensitivity power analysis was conducted using the pwr package in R [35]. Given a final sample size of *N* = 43, *α* = 0.05, and power of 0.80, our models were adequately powered to detect a medium‐to‐large independent partial effect size of *f*2 = 0.21 (equivalent to a partial *R* ^2^ ≈ 0.17) for any single predictor.

### 2.2. Measurements and Procedures

#### 2.2.1. Ultrasound

**FIGURE 1:**
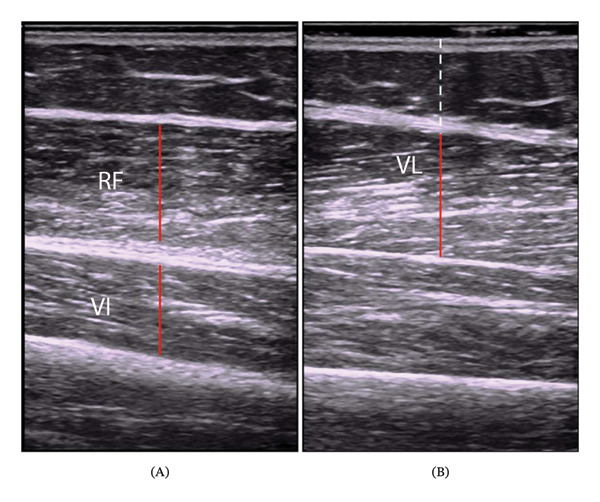
Ultrasonography images of (A) rectus femoris (RF) and vastus intermedius (VI) and (B) vastus lateralis (VL) muscles. Red lines indicate measurements of muscle thickness, while white dashed line indicates measurements of subcutaneous tissue (including fat, skin, and aponeurosis).

Ultrasound measurements were performed using 2D B‐mode ultrasonography (GE Logiq; GE Healthcare Products, United States) with a 7.5 to 12 MHz linear array frequency probe (machine settings were maintained constant for all participants: gain = 70 dB, depth = 6.0 cm, and frequency = 10 Hz). Prior to measurements, a generous amount of gel was applied to the probe, which was then gently placed on the leg by applying minimal pressure; the same evaluator performed two longitudinal ultrasound recordings at each location. All measurements were performed with the participant seated for at least 10 min with 90° knee flexion. During this period, the anterior regions of the dominant thigh were marked to identify the reference point for the ultrasound image acquisition for all muscles. For the VL markings, we first identified the greater trochanter and the lateral epicondyle of the femur and then measured the femur’s length and marked the 50% point. The medial and lateral borders of the VL were manually identified by palpation and delineated with a pen. A 50% marker was then placed to intersect with the previous marker, and the ultrasound recordings were performed at this location. The RF was identified after locating the anterior superior iliac spine and the superior border of the patella, and a 50% distance was marked to performed recordings. The VI was identified below RF recordings. Examples of US images are shown in Figure 1.

#### 2.2.2. Anthropometrics and Body Composition

Height and body mass were assessed using a stadiometer (SECA North America, Chino, CA) and digital scale (Tanita Corporation, Arlington Heights, IL). Body composition (fat mass, % body fat, and lean mass) was assessed by dual‐energy X‐ray absorptiometry (DXA) using a Lunar Prodigy densitometer (GE Healthcare, Madison, WI) [36]. All scans were analyzed using enCORE software (Version 14.0) according to manufacturer’s recommendations, and quality assurance checks were performed each testing day to ensure calibration of the DXA (average coefficient of variation [CV] over the study period was 2.95%).

#### 2.2.3. Physical Function Assessments

##### 2.2.3.1. MIVT

MIVT was assessed using a MicroFET2 hand‐held dynamometer (Hoggan Scientific LLC, Salt Lake City, Utah) [37]. Leg length was measured to calculate torque while the participant was seated upright at the edge of an exam table with 90° knee flexion. The dynamometer was then placed on the anterior part of the leg aligned with the lateral malleolus and held in place by using a belt strapped to the leg of the table in order to control for any additional resistance that could influence reliability [37]. Additionally, a marker was used to mark the location for the hand‐held dynamometer, thus maintaining consistency between trials [37]. Each participant was instructed to kick and hold as hard as possible for 3 s while maintaining bilateral hip contact with the table and crossing the arms over the chest. The highest measurement from one leg was reported in kg and converted from kg‐force meters to Newton meters (N.m). Test‐retest reliability was assessed using the interclass correlation coefficient (ICC), in which we reported a good reliability between measurements (ICC 0.86).

##### 2.2.3.2. Vertical Jump Peak Power (VJPP)

Participants were first shown the proper technique for countermovement jumps and allowed a few trials for practice (participants’ form was corrected to avoid flexing lower limb during jumps). Afterward, they were instructed to perform three VJPP with approximately 1 min of rest between trials. Jump height was evaluated using the Just Jump System (Knoxville, TN), and the averaged values were corrected using the following formula from others [38]: criterion jump height = (0.8747 × alternative jump height) – 0.0666. After correction, peak power was calculated using the following equation [39]: peak power (W) = 60.7 × [jump height (cm)] + 45.3 × [body mass (kg)] − 2055.

##### 2.2.3.3. Lower Limb Muscle Power (LLMP)

The stair‐climb test was administered as a measure of lower body muscle power. For this, participants were instructed to ascend a flight of stairs as quickly as possible (13 steps total; step height: 7 inches); no skipping of steps was allowed and at least one foot had to touch each step [40]. Participants performed two trials, and the fastest time was recorded. One minute of rest was allowed between trials. We calculated leg power (watts) following the formula [41]: [body weight (kg) × (9.8 m/s^2^) × stair height (meters)]/[time to complete stair climb (seconds)].

### 2.3. Ultrasound Data Analysis

For imaging analysis, ImageJ software, Version 1.54a (National Institutes of Health, USA) was used. After recording, all images were exported from the US machine and imported in jpeg format to another computer. Both MT and EI were assessed blindly using established methods [31]. Importantly, images from VI were taken from the images from RF (Figure 1). The average of the two ultrasound recordings performed for each location is used for analysis. All image analyses were performed by the same evaluator, who was blinded to participant identity, test condition, and functional performance results. MT: the evaluator consistently measured MT on all images by recording the distance between the superior and inferior aponeuroses of each muscle at 50% of the image size, as previously described [31]. The ICC values for MT measurements were the following: RF (0.89), VI (0.91), and VL (0.98). Interimage reliability for MT, assessed with ICC (2,1), was excellent across all muscle–region combinations across days of data collection: VL = 0.910; VI = 0.937; and VL = 0.987. EI as muscle quality: all images were displayed under identical settings for every participant to ensure consistent and precise EI analysis. The ICC values for EI measurements were the following: RF (0.93), VI (0.87), and VL (0.93). Interimage reliability for EI, assessed with ICC (2,1), was excellent for all muscle–region combinations across days of data collection: RF = 0.931; VI = 0.867; and VL 50% = 0.937. A single evaluator used the polygon tool [42] to delineate the regions of interest for the RF, VL, and VI on both images. The region of interest was delineated to include the entire visible muscle area, excluding surrounding fascia and bone, to capture the full spatial variability of grayscale values within the muscle, as previously described [31]. This approach was chosen to capture the full spatial variability of EI within the muscle. The region of interest EI values were corrected for subcutaneous thickness using the equation [43]: EI corrected = EI measured − [5.0054 × (adipose tissue)^2^] + 38.30836 × AT. We are only presenting corrected EI values, which will also be mentioned as muscle quality.

### 2.4. Statistical Analysis

Descriptive statistics for participant characteristics are presented as observed mean ± standard deviation (SD). Initially, Pearson’s bivariate correlations were evaluated to assess the unadjusted relationships between physical function assessments (MIVT, VJPP, LLMP) and the architectural parameters (MT and EI) of the VL, VI, and RF. Correlation magnitudes were classified as negligible (0.0–0.3), low (0.3–0.5), moderate (0.5–0.7), high (0.7–0.9), and very high (0.9–1.0) [44]. A small proportion of data were missing for the VI ultrasound measures (VI MT and VI EI; 4 participants, 9.3%), while all outcomes and other predictors were complete. To avoid listwise deletion and preserve statistical power, missing ultrasound values were addressed using multiple imputation by chained equations with predictive mean matching (PMM) (*m* = 20 imputations) [45, 46]. PMM was selected to preserve the observed distribution of the imputed variables and to reduce reliance on parametric assumptions. The imputation models included all analysis variables (MIVT, VJPP, LLMP; RF MT, VI MT, VL MT; RF EI, VI EI, VL EI) to preserve the joint associations among outcomes and predictors [47, 48].

Prior to interpretation, regression assumptions were strictly verified across all *m* = 20 imputed datasets using both graphical diagnostics (residuals vs fitted, Q–Q plots, scale‐location, and residuals vs leverage) and formal tests. Multicollinearity was assessed via mean variance inflation factors (VIF) and tolerance (1/VIF). Residual normality was assessed via the Shapiro–Wilk test, and homoscedasticity was assessed via the Breusch–Pagan test. In models where significant heteroscedasticity was detected (i.e., VJPP), we employed heteroscedasticity‐consistent (HC3) robust standard errors [49], computed within each imputed dataset and combined using Rubin’s rules to obtain valid pooled inference. Potentially influential observations were assessed using Cook’s distance. Some observations exceeded the heuristic threshold of 4/*n*; hence, because influence diagnostics can be sensitive in modest samples, we conducted a planned sensitivity analysis excluding the single most influential observation for each model and compared conclusions to the primary analysis.

To evaluate the independent associations between muscle morphology and physical function, we utilized multiple linear regression. Preliminary diagnostics (see the Supporting File (available here)) revealed high multicollinearity among the individual EI parameters. To ensure model stability and improve the ratio of observations to predictors, we utilized a dimension reduction strategy by creating a global quadriceps EI composite variable (the row‐mean of RF, VI, and VL EI) within each imputed dataset. Regression coefficients were estimated within each imputed dataset and combined using Rubin’s rules [50]. Model‐level fit is reported as pooled adjusted *R* ^2^ using established pooling methods for coefficients of determination. Overall model significance was evaluated using the MI‐adjusted D1 Wald test [51] (full model vs intercept‐only). The final theoretical regression model for each outcome retained four prespecified predictors: RF MT, VI MT, VL MT, and the global EI composite. To provide both clinical perspective and relative importance, we reported unstandardized (*b*) and standardized (*β*) regression coefficients with their respective 95% confidence intervals (CIs). To ensure the clinical interpretability of the intercept (representing a participant with average muscle characteristics), all continuous predictors were mean centered prior to regression analysis. All *p*‐values less than 0.05 were considered statistically significant.

## 3. Results

### 3.1. Participants’ Characteristics

A total of 43 participants (23 women/20 men; 19 White, 5 Asian, 6 Hispanic/Latino, and 13 African American) were recruited for the current study, and their characteristics are shown in Table 1.

**TABLE 1:** Participants’ characteristics.

Variable	Mean ± SD
Age (yr)	26.7 ± 8.5
Height (m)	1.69 ± 0.11
Body weight (kg)	71.7 ± 14.5
BMI (kg/m^2^)	24.8 ± 3.9
Body fat (%)	25.6 ± 9.1
Physical activity (hr/week)	7.4 + 5.7
Knee extension torque (Nm)	142.5 ± 49.6
Vertical jump peak power (W)	2175.1 ± 817.3
Lower limb muscle power (W)	518.2 ± 132.7
Muscle thickness (cm)	
Rectus femoris	2.31 ± 0.57
Vastus intermedius^∗^	1.92 ± 0.45
Vastus lateralis	2.87 ± 0.62
Echo intensity (a.u.)	
Rectus femoris	133 ± 41
Vastus intermedius^∗^	143 ± 28
Vastus lateralis	143 ± 45
Composite variable (a.u.)	
Global quadriceps echo intensity^∗^	141 ± 35

*Note:* Data are presented as observed mean ± SD.

Abbreviation: BMI, body mass index.

^∗^Contains four missing values.

Multicollinearity was observed among the anatomically related EI parameters (*r* > 0.899, VIF > 8.9), indicating substantial shared variance. Residual diagnostics for the final models are provided in the Supporting File. Assumption checks were broadly acceptable for the MIVT and LLMP models, whereas the VJPP model showed evidence of heteroscedasticity; therefore, HC3 robust standard errors were used for VJPP inference. VIF analysis and residual plots can also be found in the Supporting File. Given the presence of multicollinearity among the muscle quality variables, a composite score of all EI variables was created (global EI) and used in the multiple regression analysis.

### 3.2. MIVT

Pearson correlations between the functional outcomes and ultrasound variables are shown in the Supporting File. The fully adjusted regression model (Table 2) explained a significant proportion of the variance in MIVT, with pooled adjusted *R* ^2^ of 0.635 (95% CI [0.425, 0.784]; MI D1 omnibus test: *F* (4, 36.05) = 18.8, *p* < 0.001). In the adjusted model, RF MT (*p* = 0.009) and global EI (*p* = 0.004) were independently associated with MIVT. Greater RF thickness and lower global EI values were associated with greater torque production.

**TABLE 2:** Multiple regression analysis for maximal isometric knee extension torque (MIVT).

Variable	*b*	SE	*b* lower	*b* upper	*p* value	Beta	Beta lower	Beta upper
(Intercept)	142.534	4.571	133.265	151.803	0	NA	NA	NA
RF MT	30.736	11.08	8.252	53.22	0.009	0.349	0.094	0.603
VI MT	27.259	13.983	−1.141	55.658	0.059	0.262	−0.011	0.535
VL MT	2.158	11.336	−20.841	25.157	0.85	0.027	−0.261	0.315
Global EI	−0.522	0.17	−0.867	−0.176	0.004	−0.364	−0.605	−0.123

*Note:* Pooled adjusted *R* ^2^ = 0.635, 95% CI [0.425, 0.784]; MI D1 omnibus test: *F* (4, 36.05) = 18.8, *p* < 0.001.

Abbreviations: EI = echo intensity; MT = muscle thickness; RF = rectus femoris; VI = vastus intermedius; VL = vastus lateralis.

### 3.3. VJPP

Bivariate analyses indicated moderate to strong positive associations between VJPP and muscle morphology variables (Supporting File). The fully adjusted regression model (Table 3) presented a pooled adjusted *R* ^2^ of 0.619 (95% CI [0.406, 0.774]; MI D1 omnibus test: *F* (4, 36.1) = 17.84, *p* < 0.001). In the primary adjusted model, RF MT was the only MT variable associated with VJPP (*p* = 0.041). However, because the VJPP model showed heteroscedasticity and the identity of the significant MT predictor changed after exclusion of the most influential observation (Supporting File), attribution to a specific muscle should be interpreted cautiously. In the sensitivity analysis excluding the most influential observation, VI MT emerged as the significant thickness predictor (*p* = 0.002), indicating that the muscle‐specific VJPP finding was not fully stable in this sample (Supporting File).

**TABLE 3:** Multiple regression analysis for vertical jump peak power (VJPP).

Variable	*b*	SE	*b* lower	*b* upper	*p* value	Beta	Beta lower	Beta upper
(Intercept)	2175.143	85.723	2001.314	2348.973	0	NA	NA	NA
RF MT	523.718	246.471	23.836	1023.599	0.041	0.36	0.016	0.704
VI MT	400.276	415.327	−442.12	1242.673	0.342	0.233	−0.258	0.725
VL MT	382.971	275.278	−175.3	941.242	0.173	0.291	−0.133	0.715
Global EI	−1.797	2.598	−7.067	3.472	0.494	−0.076	−0.299	0.147

*Note:* Pooled adjusted *R* ^2^ = 0.619, 95% CI [0.406, 0.774]; MI D1 omnibus test: *F* (4, 36.1) = 17.84, *p* < 0.001.

Abbreviations: EI = echo intensity; MT = muscle thickness; RF = rectus femoris; VI = vastus intermedius; VL = vastus lateralis.

### 3.4. LLMP

Similar to other physical assessments, bivariate analyses revealed moderate to strong positive correlations between LLMP and muscle morphology (Supporting File). The fully adjusted regression model (Table 4) explained approximately 63% of the variance in LLMP (*R* ^2^ = 0.63, 95% CI [0.418, 0.781]; MI D1 omnibus test: *F* (4, 36.06) = 18.4, *p* < 0.001). For LLMP, both RF MT (*p* = 0.003) and VI MT (*p* = 0.010) were independently associated with performance in the adjusted model. Greater thickness of the RF and VI was associated with higher LLMP.

**TABLE 4:** Multiple regression analysis for lower limb muscle power (LLMP).

Variable	*b*	SE	*b* lower	*b* upper	*p* value	Beta	Beta lower	Beta upper
(Intercept)	518.239	12.308	493.281	543.196	0	NA	NA	NA
RF MT	94.347	29.924	33.612	155.083	0.003	0.4	0.142	0.657
VI MT	102.563	37.527	26.364	178.762	0.010	0.368	0.092	0.644
VL MT	41.272	30.535	−20.678	103.222	0.185	0.193	−0.097	0.483
Global EI	0.084	0.458	−0.845	1.013	0.856	0.022	−0.22	0.264

*Note:* Pooled adjusted *R* ^2^ = 0.63, 95% CI [0.418, 0.781]; MI D1 omnibus test: *F* (4, 36.06) = 18.4, *p* < 0.001.

Abbreviations: EI = echo intensity; MT = muscle thickness; RF = rectus femoris; VI = vastus intermedius; VL = vastus lateralis.

## 4. Discussion

The present study aimed to determine which ultrasound‐derived muscle characteristics of the QUAD (i.e., VL, RF, and VI; MT and EI) better explain performance in different physical assessments. In the bivariate analyses, both MT and EI were consistently correlated with all three functional outcomes, highlighting the relevance of QUAD morphology to strength and performance. We hypothesized that VL and/or VI would exhibit the strongest associations with physical performance and that MT would be a better correlate of the different tests than EI; however, this hypothesis was only partially supported. After multiple regression analysis, RF MT emerged as the primary factor associated with performance across all tests, whereas muscle quality (global EI) only significantly explained MIVT. Overall, these findings suggest that RF MT may be the most informative QUAD ultrasound measure for explaining performance in our data set.

### 4.1. MIVT

Isometric knee extension is a commonly used test under laboratory conditions; hence, understanding which muscles most influence its performance is necessary and may help to direct clinical and research interventions. Our results presented significant bivariate correlations between MIVT and all muscle variables. However, the multiple regression analysis revealed that RF MT and global EI were significant predictors of MIVT, together explaining a substantial 63.5% of the variance. These findings differ from some previous studies. One study identified VI as the greatest contributor during submaximal voluntary isometric knee extension (∼45%), followed by RF (∼22.5%), VL (∼21.5%), and VM (∼11%) [27]. Another study investigating the associations of RF and VL MT and muscle quality (e.g., EI) with MIVT found that only VL MT was a significant predictor (∼67%) [31]. In addition, other studies have reported that QUAD MT is not associated with maximal muscle strength in younger individuals [11]. VI and RF MT, as well as RF EI, have also previously been reported to influence force production during knee extension tasks [24, 30], which was not observed in the present study. Thus, there are discrepancies in findings between studies.

The contrasting findings may be explained by the lack of multivariate analyses in previous studies. To the best of the authors’ knowledge, this is one of the few studies to perform such an analysis in this context with three muscles from the QUAD. One possible explanation for RF MT emerging as the strongest predictor of MIVT is that the RF contributes substantially during isolated maximal knee extension [52]. It is well known that muscle length affects the ability of the QUAD to produce maximal torque during a maximal contraction [53, 54]; hence, RF thickness may have better reflected the QUAD muscle characteristics most closely related to torque production in the joint configuration used in the present study. In addition, because QUAD muscles act as a coordinated synergistic group and share substantial mechanical information [27], RF MT may have functioned as the most representative morphological marker in the adjusted model rather than indicating an exclusive role of the RF. These findings may be particularly relevant for future studies focusing on MIVT testing and need to evaluate on single muscle of the QUAD. Yet, global EI was also a significant predictor of MIVT. This result may reflect the fact that maximal isometric knee extension is a whole‐QUAD strength task, for which the net quality of the entire synergistic muscle group may be more relevant than the EI of any single muscle. In this context, poor muscle quality is correlated with increased intramuscular fat content [5] and increases in intramuscular fat are associated with impairments in sarcoplasmic reticulum calcium release [55], which can impair force development [56].

### 4.2. VJPP

The vertical jump test is widely applied in athletic and sports performance settings, highlighting the importance of understanding which QUAD muscles are the best predictors of this task. In the present study, the overall MT and quality were broadly related to VJPP, with only RF MT explaining nearly 62% of the variance on the multivariate analysis. To our knowledge, no previous study has examined the predictors of VJPP using a multivariate approach that simultaneously accounts for the contribution of more than two muscles from the QUAD. For instance, Jiang et al. used a multiple linear regression model to determine the strongest predictor (i.e., VL vs. RF) of jump height in elite male volleyball players [19]. In contrast to our results, they showed that the VL anatomical cross‐sectional area was the strongest predictor of jump height during the countermovement jump [19]. These differences may be partly explained by differences in the study samples, as our study included physically active men and women, whereas Jiang et al. investigated only elite male volleyball players. In addition, although MT was assessed at a similar anatomical location, Jiang et al. quantified anatomical cross‐sectional area rather than MT, which may also have contributed to the discrepant findings. Furthermore, our model included a broader set of muscles and ultrasound variables (MT and quality), which may have provided a more comprehensive representation of QUAD morphology. From a mechanistic perspective, RF may be especially relevant because it is the only biarticular muscle within the QUAD, contributing to both hip flexion and knee extension. As a result, our findings suggest that RF may play a particularly important role during the countermovement jump by helping coordinate movement and transfer force between the hip and knee joints [57]. Consistent with this, electromyographic studies report that RF activation is task‐dependent, being pronounced during knee extension while differing across squat‐type movements in which its biarticular action at the hip alters its contribution [52, 58]. Although muscle activation reflects a physiological construct distinct from the morphological measures examined here, these patterns offer complementary support for the task‐specific relevance of the RF observed in our data.

Here, we found that global EI values were not predictive of performance in the vertical jump test, whereas individual EI values from the VI and VL demonstrated moderate correlations. This finding suggests that, within a multivariate model including several muscle‐specific morphology variables, the relative contribution of each measure may depend on the extent to which it captures task‐relevant variance. In our analysis, MT from three QUAD muscles was entered into the model, which may have explained much of the variability related to muscle morphology and limited the independent contribution of the global EI index. Hence, although EI from specific muscles was associated with vertical jump performance in the bivariate analyses, these associations were not retained when EI was represented as a single global measure within the multivariate model. Previous research has shown that EI values are moderately correlated with slow‐twitch fiber percentage (*r* ^2^ = 0.642) and highly correlated with the cross‐sectional area of slow‐twitch fibers (*r* ^2^ = 0.726) [59]. In this context, the lack of an independent association between global EI and VJPP may reflect the fact that EI appears to be more closely related to muscle properties associated with oxidative or slow‐contracting characteristics, whereas vertical jump performance is primarily driven by explosive force‐generating capacity. Overall, our findings suggest that further investigation is warranted to clarify the influence of muscle quality and composition on VJPP. From a practical standpoint, although all QUAD muscles contribute to performance, targeting improvements in RF MT may be particularly beneficial, as it appears to have the greatest independent impact on vertical jump output in young physically active adults.

### 4.3. LLMP

Stair‐climb power is considered a clinically relevant functional outcome, as it reflects an individual’s ability to generate force under conditions that mimic daily activities and is a widely used clinical assessment across different populations [60, 61]. Different from the other tests, for LLMP, RF and VI MT remained significant independent predictors in the multivariate model, explaining up to 63% of the variance. These findings highlight that MT, rather than EI, is the primary determinant of power generation during the staircase test in young/middle‐aged adults. In some agreement with our results, Kleinberg et al. demonstrated that the combined MT of the RF and VL strongly predicted stair‐climb performance in professional firefighters [9]. The difference in findings from Kleinberg et al. may be explained by task and sample differences, as their study examined a weighted occupational stair‐climb task in firefighters and only included RF and VL, whereas inclusion of VI in the present model may have allowed a more direct representation of the deep knee extensor contribution. Additionally, one possible explanation for RF and VI MT emerging as independent predictors of LLMP is that stair‐climb power depends strongly on the ability of the knee extensors to generate power to elevate the body’s center of mass against gravity [62, 63]. In this context, VI may better reflect the monoarticular knee extensor contribution, whereas RF may capture additional multijoint demands of the test.

For unknown reasons, the physiological predictors of clinical assessments such as the stair‐climb test are not well explored, resulting in a limited understanding of how physiological factors influence the ability to climb stairs. One of the premises for improving performance in any task is to identify which factors should be modified, since targeted improvements in these determinants are expected to enhance overall performance [62, 63]. Thus, our study sheds light on this important issue. From a practical standpoint, these results suggest that interventions aiming to improve functional lower‐limb power should prioritize increasing the MT of the RF and VI, as both appear to play central roles during the stair‐climb power output. Importantly, although the present study focused specifically on ultrasound‐derived muscle morphology, muscle activation patterns may also contribute to performance during these tasks. Previous studies have demonstrated task‐specific differences in QUAD activation depending on joint angle [54, 64] and exercise modality [58, 65]. Therefore, future studies combining ultrasound‐derived measures of muscle morphology with EMG assessments may provide a more comprehensive understanding of how structural and neural factors interact to influence performance.

## 5. Limitations and Strengths

The limitations and strengths of this study should be acknowledged. First, our study was cross‐sectional, which prevents causal inference regarding the relationships between muscle characteristics and functional outcomes; thus, the principle that correlation does not imply causation should be kept in mind. Second, although the study had sufficient statistical power, the sample size was modest, in the context of multiple linear regression. While our sample was adequately powered for the initial bivariate correlations, our sensitivity analysis indicated that the fully adjusted regression models were powered to detect only medium‐to‐large independent effects (*f* ^2^ ≥ 0.22). Consequently, the moderate collinearity among muscle morphological variables combined with the sample size may have limited our statistical power to detect smaller, yet potentially physiologically meaningful, independent associations. Nonsignificant predictors in our final models should therefore be interpreted with caution, as type II errors cannot be entirely ruled out.

Third, while ultrasound‐derived measures of MT and EI values are reliable and widely used, muscle cross‐sectional area and intramuscular fat content obtained from MRI (or CT for intramuscular fat) are considered the gold standard. However, the EI region of interest was delineated using the entire visible muscle area to capture the overall spatial variability. Previous studies have used different approaches, including smaller or standardized sampling regions [5, 31, 43], and these methodological differences may influence EI values and limit direct comparisons across studies. Fourth, we examined three of the four QUAD muscles, with the VM not included. Previous work has estimated that the VM contributes approximately 9.5%–12.2% of total knee extensor torque [27]. Additionally, others reported a 24% reduction in torque following the removal of VM via targeted surface stimulation; however, this comparatively large contribution may, in part, reflect unintended reductions in other knee extensor muscles affected by the protocol used in the study [28]. Hence, our assumptions regarding the overall QUAD contribution to the tests performed are limited to the three muscles evaluated in this study. Fifth, only the QUAD MT and EI were evaluated; contributions from other muscle groups (e.g., hip extensors and plantar flexors) were not assessed but may also play an important role in these functional tasks. Sixth, an important advantage of the present study was that ultrasound allowed us to evaluate a deeper muscle (i.e., the VI) noninvasively, whereas assessment of this muscle with electromyography often requires invasive techniques. Lastly, the multiple regression analysis should be validated across diverse populations to determine whether the observed results are consistently replicable.

In conclusion, QUAD MT and EI are associated with overall physical function, yet their contributions differ depending on the specific task. RF MT and global EI were the variables explaining MIVT, while RF MT predicted VJPP and RF and VI MT predicted LLMP. Overall, these findings suggest that RF MT may be the most informative QUAD ultrasound measure for explaining performance in our data set. These findings highlight the nuanced roles of individual QUAD muscles and emphasize the importance of considering both MT and EI measures when evaluating muscle‐function relationships. However, generalizability is limited by the relatively small sample size and uneven ethnic distribution, despite inclusion of both sexes.

## Funding

This study was supported by the Towson University College of Health Professions Summer Undergraduate Research Institute (DFM).

## Conflicts of Interest

The authors declare no conflicts of interest.

## Supporting Information

Additional supporting information can be found online in the Supporting Information section.

## Supporting information


**Supporting Information 1** Pearson correlation matrix: pairwise Pearson correlation matrix with Holm correction. Pearson correlations of Global_EI with outcomes and MT measures. Pearson correlations of Global_EI with outcomes and MT measures, with Holm correction. Pearson correlations between functional outcomes and ultrasound variables with Holm correction. Initial pooled multicollinearity diagnostics (pre‐composite). VIF diagnostics for composite model. MIVT regression assumption verification (summary across *m* = 20 imputations). MIVT regression (composite model) excluding most influential point. VJPP regression assumption verification (summary across *m* = 20 imputations). VJPP regression (composite model; robust HC3 SEs) excluding most influential point. LLMP regression assumption verification (summary across *m* = 20 imputations). LLMP regression (composite model) excluding most influential point. Figure 1: residual diagnostics for the final composite MIVT regression model (representative imputed dataset: imputation 1). Figure 2: residual diagnostics for the final composite VJPP regression model (representative imputed dataset: imputation 1). Figure 3: residual diagnostics for the final composite LLMP regression model (representative imputed dataset: imputation 1).

**Supporting Information 2** STROBE‐checklist‐v4‐cross‐sectional.

## Data Availability

The data that support the findings of this study are available from the corresponding author upon reasonable request.
