# Supplementary material for: Quadriceps Muscle Morphology as a Marker of Performance Across Multiple Strength and Power Tests
Source: Transl Sports Med. 2026 Jul 18;2026:7599813. doi: 10.1155/tsm2/7599813 (PMC13379945; doi:10.1155/tsm2/7599813)
Supplement: Supplementary file 1 — Supporting Information 1 Pearson correlation matrix: pairwise Pearson correlation matrix with Holm correction. Pearson correlations of Global_EI with outcomes and MT measures. Pearson correlations of Global_EI with outcomes and MT measures, with Holm correction. Pearson correlations between functional outcomes and ultrasound variables with Holm correction. Initial pooled multicollinearity diagnostics (pre‐composite). VIF diagnostics for composite model. MIVT regression assumption verification (summary across m = 20 imputations). MIVT regression (composite model) excluding most influential point. VJPP regression assumption verification (summary across m = 20 imputations). VJPP regression (composite model; robust HC3 SEs) excluding most influential point. LLMP regression assumption verification (summary across m = 20 imputations). LLMP regression (composite model) excluding most influential point. Figure 1: residual diagnostics for the final composite MIVT regression model (representative imputed dataset: imputation 1). Figure 2: residual diagnostics for the final composite VJPP regression model (representative imputed dataset: imputation 1). Figure 3: residual diagnostics for the final composite LLMP regression model (representative imputed dataset: imputation 1). [file TSM2-2026-7599813-s001.docx]

Pearson correlation matrix.

|  | VJPP | LLMP | MIVT | RF_MT | VI_MT | VL_MT | RF_EI | VI_EI | VL_EI |
| --- | --- | --- | --- | --- | --- | --- | --- | --- | --- |
| VJPP | 1.000 | 0.856 | 0.837 | 0.609 | 0.601 | 0.628 | -0.335 | -0.554 | -0.519 |
| LLMP | 0.856 | 1.000 | 0.804 | 0.569 | 0.651 | 0.561 | -0.269 | -0.543 | -0.440 |
| MIVT | 0.837 | 0.804 | 1.000 | 0.574 | 0.620 | 0.526 | -0.494 | -0.694 | -0.651 |
| RF_MT | 0.609 | 0.569 | 0.574 | 1.000 | 0.450 | 0.514 | -0.248 | -0.416 | -0.435 |
| VI_MT | 0.601 | 0.651 | 0.620 | 0.450 | 1.000 | 0.591 | -0.427 | -0.491 | -0.510 |
| VL_MT | 0.628 | 0.561 | 0.526 | 0.514 | 0.591 | 1.000 | -0.496 | -0.306 | -0.621 |
| RF_EI | -0.335 | -0.269 | -0.494 | -0.248 | -0.427 | -0.496 | 1.000 | 0.501 | 0.899 |
| VI_EI | -0.554 | -0.543 | -0.694 | -0.416 | -0.491 | -0.306 | 0.501 | 1.000 | 0.621 |
| VL_EI | -0.519 | -0.440 | -0.651 | -0.435 | -0.510 | -0.621 | 0.899 | 0.621 | 1.000 |

*Note:* For descriptive purposes, correlations were computed observed values among complete cases.

Pairwise Pearson correlation matrix with Holm correction.

|  | VJPP | LLMP | MIVT | RF_MT | VI_MT | VL_MT | RF_EI | VI_EI | VL_EI |
| --- | --- | --- | --- | --- | --- | --- | --- | --- | --- |
| VJPP | 1.000 | 0.856* | 0.837* | 0.609* | 0.601* | 0.628* | -0.335 | -0.554* | -0.519* |
| LLMP | 0.856* | 1.000 | 0.804* | 0.569* | 0.651* | 0.561* | -0.269 | -0.543* | -0.440* |
| MIVT | 0.837* | 0.804* | 1.000 | 0.574* | 0.620* | 0.526* | -0.494* | -0.694* | -0.651* |
| RF_MT | 0.609* | 0.569* | 0.574* | 1.000 | 0.450* | 0.514* | -0.248 | -0.416* | -0.435* |
| VI_MT | 0.601* | 0.651* | 0.620* | 0.450* | 1.000 | 0.591* | -0.427* | -0.491* | -0.510* |
| VL_MT | 0.628* | 0.561* | 0.526* | 0.514* | 0.591* | 1.000 | -0.496* | -0.306 | -0.621* |
| RF_EI | -0.335 | -0.269 | -0.494* | -0.248 | -0.427* | -0.496* | 1.000 | 0.501* | 0.899* |
| VI_EI | -0.554* | -0.543* | -0.694* | -0.416* | -0.491* | -0.306 | 0.501* | 1.000 | 0.621* |
| VL_EI | -0.519* | -0.440* | -0.651* | -0.435* | -0.510* | -0.621* | 0.899* | 0.621* | 1.000 |

*Note:* For descriptive purposes, correlations were computed using observed values among complete cases. Asterisks indicate correlations with Holm-adjusted *p* < 0.05.

Pearson correlations of Global_EI with outcomes and MT measures.

|  | VJPP | LLMP | MIVT | RF_MT | VI_MT | VL_MT |
| --- | --- | --- | --- | --- | --- | --- |
| Global_EI | -0.509 | -0.446 | -0.67 | -0.401 | -0.527 | -0.554 |

*Note:* For descriptive purposes, correlations were computed using observed values among complete cases.

Pearson correlations of Global_EI with outcomes and MT measures, with Holm correction.

Variable Correlation VJPP -0.509*

LLMP -0.446*

MIVT -0.670* RF_MT -0.401* VI_MT -0.527*

VL_MT -0.554*

*Note:* For descriptive purposes, correlations were computed using observed values among complete cases. Asterisks indicate correlations with Holm-adjusted *p* < 0.05.

Pearson correlations between functional outcomes and ultrasound variables with Holm correction.

|  | RF_MT | VI_MT | VL_MT | RF_EI | VI_EI | VL_EI |
| --- | --- | --- | --- | --- | --- | --- |
| VJPP | 0.609* | 0.601* | 0.628* | -0.335 | -0.554* | -0.519* |
| LLMP | 0.569* | 0.651* | 0.561* | -0.269 | -0.543* | -0.440* |
| MIVT | 0.574* | 0.620* | 0.526* | -0.494* | -0.694* | -0.651* |

*Note:* Correlations were computed using observed values among complete cases. Holm correction was applied across the 18 outcome-by-ultrasound correlations. Asterisks indicate Holm-adjusted *p* < 0.05.

Initial Pooled Multicollinearity Diagnostics (Pre-Composite).

| Predictor | Mean VIF | Min | Max | Mean Tolerance |
| --- | --- | --- | --- | --- |
| RF_EI | 5.762 | 5.637 | 5.905 | 0.174 |
| RF_MT | 2.136 | 1.993 | 2.338 | 0.469 |
| VI_EI | 2.115 | 1.818 | 2.356 | 0.476 |
| VI_MT | 2.134 | 1.896 | 2.327 | 0.470 |
| VL_EI | 8.914 | 8.280 | 9.672 | 0.112 |
| VL_MT | 2.824 | 2.657 | 3.049 | 0.355 |

VIF Diagnostics for Composite Model.

| Predictor | Mean VIF | Min | Max | Mean Tolerance |
| --- | --- | --- | --- | --- |
| Global_EI_c | 1.604 | 1.491 | 1.696 | 0.624 |
| RF_MT_c | 1.782 | 1.702 | 1.838 | 0.562 |
| VI_MT_c | 1.985 | 1.759 | 2.133 | 0.505 |
| VL_MT_c | 2.285 | 2.188 | 2.375 | 0.438 |

MIVT Regression Assumption Verification (Summary across m=20 imputations).

| Metric | | Min | | | | | Max | | Mean | |
| --- | --- | --- | --- | --- | --- | --- | --- | --- | --- | --- |
| Normality (Shapiro-Wilk p-value) | | | | | 0.723 | | 0.984 | | 0.887 | |
| Homoscedasticity (Breusch-Pagan p-value) | | | | | | 0.168 | 0.535 | | 0.346 | |
| Influence (Max Cook’s D) | | | | 0.162 | | | 0.237 | | 0.188 | |
| Influence (N with Cook’s D > 4/n) | | | | | 5.000 | | 7.000 | | 5.450 | |
|  | MIVT Regression (Composite Model) Excluding Most Influential Point. | | | | | | | | |  |
| term | b | SE | b Lower | b Upper | p-val | Beta | | Beta Lower | | Beta Upper |
| (Intercept) | 144.729 | 4.360 | 135.879 | 153.580 | 0.000 | NA | | NA | | NA |
| RF_MT_c | 27.464 | 10.572 | 5.983 | 48.944 | 0.014 | 0.317 | | 0.069 | | 0.565 |
| VI_MT_c | 25.366 | 13.263 | -1.610 | 52.342 | 0.064 | 0.252 | | -0.017 | | 0.520 |
| VL_MT_c | -2.472 | 10.879 | -24.566 | 19.623 | 0.822 | -0.032 | | -0.313 | | 0.250 |
| Global_EI_c | -0.632 | 0.167 | -0.972 | -0.292 | 0.001 | -0.459 | | -0.706 | | -0.211 |

VJPP Regression Assumption Verification (Summary across m=20 imputations).

| Metric | | Min | | | | Max | Mean | |
| --- | --- | --- | --- | --- | --- | --- | --- | --- |
| Normality (Shapiro-Wilk p-value) | | | | | 0.302 | 0.612 | 0.464 | |
| Homoscedasticity (Breusch-Pagan p-value) | | | | | 0.002 | 0.005 | 0.003 | |
| Influence (Max Cook’s D) | | | | 0.521 | | 0.730 | 0.646 | |
| Influence (N with Cook’s D > 4/n) | | | | | 3.000 | 3.000 | 3.000 | |
| VJPP Regression (Composite Model; Robust HC3 SEs) Excluding Most Influential Point. | | | | | | | | |
| term | b | SE | b Lower | b Upper | p-val | Beta | Beta Lower | Beta Upper |
| (Intercept) | 2157.618 | 75.036 | 2005.309 | 2309.926 | 0.000 | NA | NA | NA |
| RF_MT_c | 372.207 | 198.616 | -31.446 | 775.861 | 0.070 | 0.258 | -0.022 | 0.539 |
| VI_MT_c | 764.766 | 222.694 | 311.907 | 1217.624 | 0.002 | 0.416 | 0.167 | 0.666 |
| VL_MT_c | 321.243 | 258.750 | -204.147 | 846.633 | 0.223 | 0.246 | -0.156 | 0.648 |
| Global_EI_c | -1.763 | 2.567 | -6.975 | 3.449 | 0.497 | -0.075 | -0.295 | 0.146 |

LLMP Regression Assumption Verification (Summary across m=20 imputations).

| Metric | | Min | | | | | Max | | Mean | |
| --- | --- | --- | --- | --- | --- | --- | --- | --- | --- | --- |
| Normality (Shapiro-Wilk p-value) | | | | | 0.400 | | 0.894 | | 0.620 | |
| Homoscedasticity (Breusch-Pagan p-value) | | | | | | 0.040 | 0.094 | | 0.072 | |
| Influence (Max Cook’s D) | | | | 0.317 | | | 0.352 | | 0.331 | |
| Influence (N with Cook’s D > 4/n) | | | | | 3.000 | | 5.000 | | 3.800 | |
|  | LLMP Regression (Composite Model) Excluding Most Influential Point. | | | | | | | | |  |
| term | b | SE | b Lower | b Upper | p-val | Beta | | Beta Lower | | Beta Upper |
| (Intercept) | 511.420 | 11.780 | 487.508 | 535.332 | 0.000 | NA | | NA | | NA |
| RF_MT_c | 114.863 | 29.634 | 54.660 | 175.066 | 0.000 | 0.517 | | 0.246 | | 0.788 |
| VI_MT_c | 89.665 | 36.269 | 15.892 | 163.439 | 0.019 | 0.332 | | 0.056 | | 0.608 |
| VL_MT_c | 11.717 | 31.575 | -52.409 | 75.843 | 0.713 | 0.055 | | -0.245 | | 0.354 |
| Global_EI_c | -0.132 | 0.444 | -1.034 | 0.770 | 0.768 | -0.037 | | -0.286 | | 0.213 |

# Maximal Isometric Knee Extension Torque (MIVT)

Residuals vs Fitted Q−Q Residuals


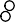

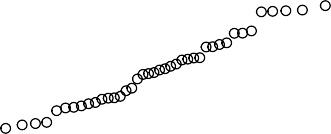


Residuals

0

50

Standardized residuals

0 1 2

100 150 200 −2 −1 0 1 2

Fitted values Theoretical Quantiles

−50

−2

Scale−Location Residuals vs Leverage

0.5


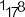

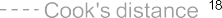


Standardized residuals

1.0

1.5

Standardized residuals

0 1 2

0.5

0.0

0.5

−2

100 150 200 0.00 0.05 0.10 0.15 0.20 0.25

Fitted values Leverage

Figure 1: Residual diagnostics for the final composite MIVT regression model (representative imputed dataset: imputation 1).

# Vertical Jump Peak Power (VJPP)

Residuals vs Fitted Q−Q Residuals


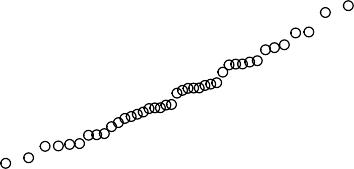


Residuals

0

500

1500

Standardized residuals

−1 0 1 2 3

1000 1500 2000 2500 3000 3500 −2 −1 0 1 2

Fitted values Theoretical Quantiles

−1000

Scale−Location Residuals vs Leverage


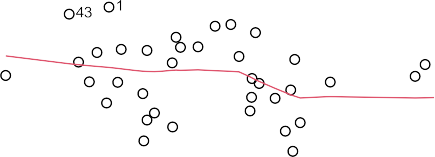


Standardized residuals

1.5

Standardized residuals

0 1 2 3

1


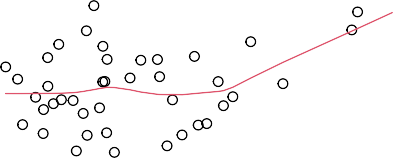


Cook's distance

0.5

1.0

1000 1500 2000 2500 3000 3500 0.00 0.05 0.10 0.15 0.20 0.25

0.0

0.5

−2

Fitted values Leverage

Figure 2: Residual diagnostics for the final composite VJPP regression model (representative imputed dataset: imputation 1).

# Lower Limb Muscle Power (LLMP)

Residuals vs Fitted Q−Q Residuals


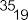

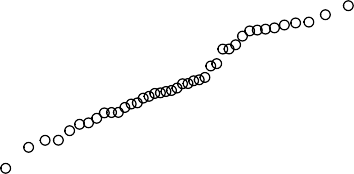


Residuals

0

100

Standardized residuals

−2 −1 0

1

2

300 400 500 600 700 −2 −1 0 1 2

Fitted values Theoretical Quantiles

−200

Scale−Location Residuals vs Leverage


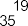

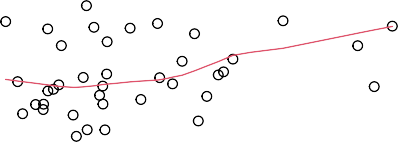


Cook's distance ^19^

1.5

0.5

Standardized residuals

1.0

Standardized residuals

0 1 2

0.5

0.0

0.5

−2

300 400 500 600 700 0.00 0.05 0.10 0.15 0.20 0.25

Fitted values Leverage

Figure 3: Residual diagnostics for the final composite LLMP regression model (representative imputed dataset: imputation 1)
